# Prevalence and Genetic Diversity of *Toxoplasma gondii* in Free-Ranging Chickens from the Caribbean

**DOI:** 10.2478/s11686-019-00071-7

**Published:** 2019-05-20

**Authors:** C. M. Hamilton, R. Robins, R. Thomas, C. Oura, S. Oliveira, I. Villena, E. A. Innes, F. Katzer, P. J. Kelly

**Affiliations:** 1 Moredun Research Institute, Pentlands Science Park, Midlothian, EH26 0PZ UK; 2 School of Veterinary Medicine, Ross University, Island Main Road, West Farm, Saint Kitts and Nevis; 3 Pioneer Kennels and Veterinary Clinic, St. Johns, Antigua and Barbuda; 4 Livestock Development Unit, Ministry of Agriculture and Environment, Botanical Gardens, Roseau, Dominica; 5 Department of Basic Veterinary Sciences, School of Veterinary Medicine, The University of the West Indies, Eric Williams Medical Sciences Complex, Mount Hope, Trinidad and Tobago; 6 Department of Preventive Veterinary Medicine and Animal Health, School of Veterinary Medicine, University of São Paulo, São Paulo, Brazil; 7 Laboratory of Parasitology, National Reference Centre on Toxoplasmosis, EA 7510, University Reims Champagne Ardenne and Hospital Maison Blanche, CHU Reims, Reims, France

**Keywords:** *Toxoplasma gondii*, Chickens, Antigua and Barbuda, Dominica, Trinidad and Tobago

## Abstract

**Purpose:**

*Toxoplasma gondii* is a zoonotic parasite capable of infecting a wide range of hosts. Free-range chickens are important sentinels in the epidemiology of this parasite as they feed from the ground and are likely to ingest oocysts shed in the faeces of infected cats. Atypical strains of *T. gondii* are known to dominate in South America where they are associated with more severe disease in humans, yet relatively little is known about the strains circulating in neighbouring Caribbean islands.

**Methods:**

In this study, hearts and brains were collected from free-range chickens in Antigua and Barbuda (*n* = 45), Dominica (*n* = 76) and Trinidad (*n* = 41), and DNA was extracted for nested ITS1 PCR and PCR–RFLP. Sera were collected and screened for antibodies using the modified agglutination test (MAT).

**Results:**

Antibodies to *T. gondii* were detected in 20.5, 38.2 and 17.1% of chickens in Antigua and Barbuda, Dominica and Trinidad, respectively. *Toxoplasma gondii* DNA was also detected by PCR in 24.4, 17.1 and 17.1% of chickens, respectively, giving an overall prevalence of 31.1, 42.1, and 29.3% for each of the 3 island nations. Results of PCR–RFLP revealed 2 new atypical genotypes (designated ToxoDB #281 and #282) and one Type III (ToxoDB #2) in chickens from Antigua. Partial genotyping of a further 8 isolates (7 from Antigua and one from Trinidad) revealed different allele-types at five or more markers for 7 of the isolates, suggesting atypical genotypes.

**Conclusions:**

This is the first study to report the prevalence of *T. gondii* in free-range chickens in Antigua and Barbuda, Dominica and Trinidad and Tobago. It is also the first to report the presence of atypical genotypes in Antigua and Barbuda and Trinidad and Tobago.

## Introduction

The zoonotic protozoan parasite, *Toxoplasma gondii*, is ubiquitous worldwide where it can cause infection in almost all warm-blooded animals. Felids are the only known definitive hosts capable of shedding oocysts in their faeces and contaminating the environment [30]. In humans, transmission routes of the parasite include ingestion of sporulated oocysts from water or unwashed contaminated food, ingestion of tissue cysts in raw or undercooked infected meat, or vertical transmission from mother to foetus during a primary infection. Chickens are important hosts in the epidemiology of *T. gondii* because they feed from the ground and are likely to ingest oocysts, making them good sentinels for environmental contamination. In 2002, a survey of *T. gondii* in free-roaming chickens was initiated with the goal of characterising the genetic diversity of *T. gondii* on a global basis [14, 18]. Consequently, free-ranging chickens are widely used to study the prevalence and genetic variation of *T. gondii* worldwide [10, 36–38].

Previous research has shown that isolates of *T. gondii* from Central and South America are genetically distinct from the clonal lineages that dominate in North America and Europe [3, 33], and they are more associated with severe disease, even in immune competent people [8]. Studies in the Caribbean have demonstrated a high seroprevalence of *T. gondii* in livestock [9, 27, 28], chickens [10, 29], cats [21] and dogs [22, 23] and that atypical strains are more common than previously thought [29]. Seroprevalence data for humans in the region are lacking; however, a recent study involving 10 Caribbean islands revealed that pregnant women in Antigua and Barbuda, and Dominica had some of the highest seroprevalence rates recorded in the study [23]. It is not known what genotypes are circulating on these islands or how widespread *T. gondii* oocyst contamination is; therefore, the aim of this study was to use free-ranging chickens to investigate the prevalence and genetic diversity of *T. gondii* on four Caribbean islands.

## Materials and Methods

### Sampling Locations

Antigua and Barbuda is a twin island country (440 km^2^) in the Leeward Islands in the Eastern Caribbean (17°5′N, 61°46′W) with a population of approximately 101,000. Until 2017, over 1600 people inhabited Barbuda; however, following hurricane Irma the island is now virtually uninhabited. They are low-lying islands with an average annual rainfall of 990 mm and an average temperature of 26.7 °C. Dominica (761 km^2^) is in the Windward Islands in the Eastern Caribbean (15°25′N, 61°18′W) and has a population of approximately 73,500. The island is more mountainous with tropical rainforests and an average annual rainfall of 9000 mm and an average temperature of 26.3 °C. Trinidad and Tobago is a twin island country in the Windward Islands (10°26′N, 61°18′W) with a population of approximately 1.4 million. Trinidad is the larger island (4768 km^2^) and has an average annual rainfall of 1891 mm and an average temperature of 25.9 °C.

### Animals and Ethical Approval

Chickens (*Gallus gallus domesticus*) were collected from four different Caribbean islands (representing 3 island nations) following ethical approval from the Institutional Animal Care and Use Committee at Ross University School of Veterinary Medicine (Project Submission 16-5-011). Forty-five free-ranging chickens in Antigua and Barbuda (collected April 2016) and seventy-six free-ranging chickens in Dominica (collected August–October 2016) were collected from each parish around the island with permission from the Chief Veterinary Officers on each island (Dr. Tubal Edwards and Dr. Reginald Thomas, respectively). In Trinidad, forty-one chickens were collected from five backyard poultry farms as part of a separate study [6].

### Sample Collection and Processing

Following humane euthanasia, blood (for serum isolation), heart and brain were collected from each chicken and processed as described previously [29]. Sera were sent to the Toxoplasma National Reference Centre (Reims, France) to be tested for antibodies to *T. gondii* using a modified agglutination test [17, 20, 34]. The MAT antigen was prepared based on previously described methods [13, 17]. An antibody titre of greater than or equal to 1:6 was considered positive for exposure to *T. gondii*. DNA extraction was performed on 400 μl of digested brain and heart homogenate per chicken and screened for *T. gondii* using a nested PCR targeting the ITS1 region between the 18S and 5.8S rRNA genes, as previously described [7, 28, 29]. Any samples which were positive by ITS1 PCR were genotyped using a multiplex nested PCR–RFLP targeting 10 genetic markers, as previously described [28, 29].

### Statistical Analysis

Level of agreement between MAT results and ITS1 PCR results was investigated using Chi-square test of association and Cohen’s kappa coefficient. Positive agreement (PA), negative agreement (NA) and the proportion of overall agreement (p_o_) between the two tests were also calculated as follows*: PA *=* 2a*/(*2a *+* b *+ *c*), *PN *=* 2d*/(*2d *+* b *+ *c*), *po *=* a *+* d*/(*a *+* b *+ *c *+* d*), with *a, b, c, d* referring to the positive and negative cell values of the 2 × 2 contingency table [5, 32]. For Chi-square analysis, a *P* value of < 0.05 was deemed significant. For Cohen’s kappa coefficient, the strength of agreement was based on the following categories: < 0.01 = poor agreement; 0.01–0.20 = slight agreement; 0.21–0.40 = fair agreement; 0.41–0.60 = moderate agreement; 0.61–0.80 = substantial; 0.81–1.0 = almost perfect agreement [31].

## Results

### Molecular and Serological Detection of *T. gondii* in Chickens

Chickens positive for *T. gondii* by MAT and/or PCR were found on all islands (Table 1): 14 out of 45 chickens (31.1%) in Antigua and Barbuda, 32 out of 76 chickens (42.1%) in Dominica and 12 out of 41 chickens (29.3%) in Trinidad. Five chickens from Antigua and Barbuda were positive by PCR, but negative by serology, and 3 chickens were positive by serology, but negative by PCR. In Dominica, 3 chickens were positive by PCR, but negative by serology, and 19 chickens were positive by serology, but negative by PCR. In Trinidad, 5 chickens were positive by PCR but tested negative by MAT, and 4 chickens tested positive by MAT but were negative by PCR (Table 2). Despite the apparent discrepancy between MAT and PCR results, Chi-square analysis revealed a significant association between results of the two tests (χ^2^ = 17.29, *d.f*. = 1, *P* < 0.001). Binomial analysis revealed a high overall agreement between the tests (Table 3); however, Cohen’s kappa coefficient demonstrated there was only a fair level of agreement between positive MAT results and positive PCR results (*κ* = 0.3182).

**Table 1:** *Toxoplasma gondii* detection by serology and PCR in chickens from Antigua and Barbuda, Dominica and Trinidad

Caribbean nation	No. of chickens	MAT titre^a^								No. of seropositive (%)	No. of PCR-positive (%)	Total no. of positive chickens^b^ (%)
		≤1:3	1:6	1:10	1:25	1:50	1:100	1:200	1:800			
Antigua and Barbuda	45	35	2	0	5	1	0	0	1	9 (20.5%^c^)	11 (24.4%)	14 (31.1%)
Dominica	76	47	5	1	12	6	2	2	1	29 (38.2%)	13 (17.1%)	32 (42.1%)
Trinidad and Tobago	41	34	1	0	4	0	1	1	0	7 (17.1%)	7 (17.1%)	12 (29.3%)

^a^MAT titre of ≥ 1:6 was deemed seropositive

^b^Chickens positive either by MAT and/or PCR

^c^Serology for Antigua and Barbuda is based on 44 samples as no serum could be isolated from one chicken

**Table 2:** Contingency table demonstrating results of the serological and molecular detection methods for *Toxoplasma gondii* from free-roaming chickens from all islands

	MAT		
	POS	NEG	Total
PCR			
POS	18	27	45
NEG	13	103	116
Total	31	130	161

**Table 3:** Level of agreement between results obtained by MAT and PCR

	Positive agreement (PA)	Negative agreement (NA)	Overall agreement (p_o_)
MAT versus PCR	0.47	0.84	0.75

### Genetic Characterization of *T. gondii* in Chickens

A full PCR–RFLP genotype could not be obtained for all samples due to lack of amplification at some of the markers. Of the 11 chickens from Antigua and Barbuda which tested positive for *T. gondii* by ITS1 PCR, 10 were successfully genotyped at 6 or more markers and the majority appears to have atypical genotypes (Table 4). Two of the genotypes are new and have not been previously described (TgCkAn14, designated ToxoDB #281, and TgCkAn18, designated ToxoDB #282; Chunlei Su, personal communication). Isolate TgCkAn19 had a clonal Type III genotype (ToxoDB #2) and isolate TgCkAn33 had Type III alleles at 8 out of 10 markers indicating a possible clonal Type III genotype. Only 4 of the 13 ITS1 PCR-positive chickens from Dominica could be partially genotyped, but amplification was achieved at too few markers so data are excluded. Only one of the 7 ITS1 PCR-positive chickens from Trinidad could be partially genotyped at 5 markers (Table 4). Of those samples where amplification was achieved at 5 or more markers, the genotypes appear atypical.

**Table 4:** Genetic characterisation of *Toxoplasma gondii* isolated from chickens in Antigua and Barbuda (TgCkAn), Dominica (TgCkDom) and Trinidad (TgCkTri)

DNA Isolate ID^a^	MAT titre	Genetic characterisation											
		SAG1	SAG2 (5′ and 3′)	Alt. SAG2	SAG3	BTUB	GRA6	C22-8	C29-2	L358	PK1	Apico	Possible ToxoDB RFLP Genotype
TgCkAn03	1:25	I	I	na	I	I	III	II	na	III	I	na	#13 or #78
TgCkAn12	1:3	I	I	I	III	III	III	II	III	III	I	na	Most likely #282
TgCkAn14	1:3	I	I	I	III	I	II	III	III	I	II	III	#281 (new)
TgCkAn17	1:25	I	I	I	III	III	III	II	III	III	I	na	Most likely #282
TgCkAn18	1:3	I	I	I	III	III	III	II	III	III	I	III	#282 (new)
TgCkAn19	< 1:3	II or III	III	III	III	III	III	III	III	III	III	III	#2
TgCkAn28	1:25	I	I	na	na	III	III	II	na	I	na	na	#95, #96, #97, #108, #152, #240 or #251
TgCkAn29	1:3	I	I	na	III	III	III	na	na	III	I	na	#87, #88, #90 or #282
TgCkAn33	1:6	II or III	III	na	III	III	III	III	na	III	III	na	Possibly #2
TgCkAn36	1:25	I	I	na	III	III	III	II	na	III	I	na	#282 or #88
TgCkTri05	< 1:3	na	III	na	II	I	III	na	na	na	na	III	Possibly new

^a^*An* Antigua and Barbuda, *Dom* Dominica, *Tri* Trinidad, *na* not amplified

## Discussion

The results of this study demonstrate a high exposure to non-clonal genotypes of *T. gondii* in free-ranging and backyard chickens in the Caribbean. Although the sample sizes were not large, chickens were collected from different locations around each island representing most parishes. Seroprevalence results in the present study are consistent with previous studies in the Caribbean that reported seroprevalences of 32% in free-ranging chickens in St. Kitts [29] and 26.9% in Grenada [10]. They are also comparable to studies on chickens in Indonesia (24.4%), Poland (30%) and Vietnam (24.2%) [19]. Seroprevalence in chickens in Central and South America is high, ranging from 38 to 66% in South America and 74–85.7% in Central America [14]. Free-range chickens are important indicators of environmental contamination with *T. gondii* oocysts since they feed from the ground and are thus very likely to come into contact with infected cat faces. The results of the present study strongly suggest that the environments in Antigua and Barbuda, Dominica and Trinidad are contaminated with *T. gondii* oocysts shed by infected cats. Indeed, previous studies of herbivorous livestock in Dominica and Trinidad demonstrated *T. gondii* seroprevalences of 67.3% and 35.7%, respectively, in sheep and 58.1% and 42.9%, respectively, in goats, further attesting to environmental contamination since ingestion of oocysts on contaminated pastures is the main transmission route for these animals [1, 27]. There are very few data on the prevalence of *T. gondii* in humans in the Caribbean. In a recent study of pregnant women from ten Caribbean islands, anti-*Toxoplasma* IgG was detected in 32% (12 out of 38) of women from Antigua and Barbuda, and 59% (29 out of 49) of women from Dominica [23]. A study of 504 cord blood samples taken from newborns at two hospitals in Trinidad revealed a *T. gondii* seroprevalence of 43.7% and a questionnaire survey revealed that the highest seroprevalences were in neonates from mothers who owned a cat, practiced outdoor gardening or consumed raw meat [2]. These high levels of human exposure are consistent with the high environmental contamination indicated by the present study and high infection rates in food animals demonstrated in previous studies [1, 28].

Unfortunately, a full PCR–RFLP genotyping profile was not achieved for all isolates due to lack of amplification at the single-copy markers. This is a common problem in *Toxoplasma* research [11] and can only be rectified with the use of a mouse bioassay to amplify the parasites to sufficient levels to achieve complete genotyping, which is not always possible. Although a full PCR–RFLP genotyping profile was not achieved for all samples in the present study, amplification at five or more loci was possible for 11 of the DNA isolates and allowed for the demonstration of a predominance of non-clonal genotypes (i.e., different allele-types at different markers for the same isolate). With the exception of one isolate which had a Type III clonal lineage (TgCkAn19) and one which appeared to have a Type III clonal lineage (TgCkAn33) all of the isolates appeared to be atypical—two of which are new and have not been previously described (#281 and #282). Isolates TgCkAn12 and 17 appear to have a similar genetic profile to the new genotype #282. Isolate TgCkAn03 has a similar profile to ToxoDB RFLP genotype #13 which has previously been isolated from chickens in St. Kitts [29] and Grenada [10] and has been associated with severe disease in humans [4, 29]. Our results appear to be consistent with previous studies in St. Kitts which have demonstrated a predominance of atypical genotypes in free-ranging chickens [29] and the presence of atypical genotypes in cats [21]. However, our results are in contrast to what is observed on the fellow Caribbean island of Grenada where studies in free-range chickens [10, 24], dogs [22] and rats [16] have demonstrated a dominance of the Type III clonal lineage. *Toxoplasma gondii* was previously thought to have a clonal population structure comprising three dominant lineages (Types I, II, III) with very little genetic diversity. However, it is now known that the structure is much more diverse and atypical strains have been reported worldwide, with the majority being reported in Central and South America [35]. The majority of studies on free-roaming chickens in South America have been conducted in Brazil and have demonstrated a predominance of atypical genotypes with few clonal genotypes isolated [12, 15, 25, 37].

From the present study, and previous studies [29], it would appear that the genetic diversity of *T. gondii* in some islands of the Caribbean is similar to that of South America. However, to determine the exact genotypes which are present on the islands in this study, a further study would be needed involving a mouse bioassay and isolation of viable parasites which would allow for full PCR–RFLP profiling.

In this study there was a fair to moderate level of agreement between the MAT results and PCR results. It has been shown previously that *T. gondii* can be isolated from seronegative chickens [24, 29] indicating that perhaps the MAT is not appropriate for detecting early infection when levels of IgM are high and IgG are low (and, therefore, undetectable by MAT). A recent study validating the performance of different serological tests in experimentally infected chickens highlighted that the MAT could only consistently detect antibodies in those animals which had been inoculated with ≥ 10^4^*T. gondii* oocysts [34]. Furthermore, the results were dependent on the inoculating strain—with positive MAT results being detected for chickens infected with 10^3^ oocysts of the ME49 strain (Type II) but not for chickens infected with 10^3^ oocysts of CZ-Tiger strain (Type II) or NED strain (Type III). In the present study there were a number of chickens positive by MAT yet negative by PCR, including one with a titre of 1:800 and two with a titre of 1:200. False negative PCR results could be due to the inhomogeneous distribution of cysts in the digested tissues and the smaller volume of homogenate used for DNA extraction (versus bioassay where all of the homogenate would be used). Chickens are clinically resistant to *T. gondii* so the discrepancy may also be due to a lack of cysts in the tissues [26].

In conclusion, the results of the present study demonstrate the presence of atypical genotypes of *T. gondii* in Antigua and Barbuda and Trinidad and Tobago and that chickens on these islands, as well as on Dominica, are commonly exposed to the parasite, indicating widespread environmental contamination with oocysts. Taken with other data from the region, our study should alert health workers to the presence of *T. gondii* and stimulate public health campaigns urging people, particularly pregnant women, to avoid infections by carefully washing their hands after working with soil, thoroughly cooking their meat and washing fruit and vegetables before consumption.
